# Sex differences in the aging pattern of renin–angiotensin system serum peptidases

**DOI:** 10.1186/s13293-017-0128-8

**Published:** 2017-02-03

**Authors:** A. Fernández-Atucha, A. Izagirre, A. B. Fraile-Bermúdez, M. Kortajarena, G. Larrinaga, P. Martinez-Lage, E Echevarría, J. Gil

**Affiliations:** 1 Department of Nursing I, School of Nursing, University of the Basque Country (UPV/EHU), P.O. Box 699, E-48080 Bilbao, Bizkaia Spain; 2 Department of Neurology, CITA-Alzheimer Foundation, San Sebastian, Spain; 3 Centro de Investigación Biomédica en Red de Salud Mental (CIBERSAM), Madrid, Spain; 4 Department of Physiology, Faculty of Medicine, University of the Basque Country (UPV/EHU), P.O. Box 699, E-48080 Bilbao, Bizkaia Spain

**Keywords:** Sex differences, Renin–angiotensin system, Serum peptidases, Aging

## Abstract

**Background:**

Serum peptidases, such as angiotensin-converting enzyme (ACE), angiotensin-converting enzyme-2 (ACE2), neutral endopeptidase (NEP), aminopeptidase N (APN), and aminopeptidase A (APA), are important elements of the renin–angiotensin system (RAS). Dysregulation of these enzymes has been associated with hypertension and cardiovascular risk. In the present study, serum activities of RAS peptidases were analyzed to evaluate the existence of sexual differences, with a possible different pattern in pre- and post-andropausal/post-menopausal participants.

**Methods:**

One hundred and eighteen healthy men and women between 41 and 70 years of age (58 women and 60 men) were recruited to participate in the study. Serum RAS-regulating enzymes were measured by spectrofluorimetry. Enzymatic activity was recorded as units of enzyme per milliliter of serum (U/mL).

**Results:**

Significantly lower serum APA activity was observed in men with respect to women; no sex differences were detected for ACE, ACE2, NEP, or APN. Significantly lower APA and ACE serum activity were observed in older men compared to older women. In contrast, younger (<55 years) men had significantly higher values of NEP serum activity than younger women. Significantly lower ACE serum activity was detected in older men compared to younger men. In women, significantly higher ACE2 serum activity was observed in older women compared to younger women.

**Conclusions:**

These results suggest a differential effect of aging on the activity of RAS enzymes in men and women, especially with respect to the breakpoint of andropausia/menopausia, on the critical serum enzymatic activities of the RAS, which could correlate with sexual differences in cardiovascular risk.

## Background

The renin–angiotensin system (RAS) has been classically described as a circulating hormone system that regulates hydroelectrolytic balance and cardiovascular function [1]. The better-known bioactive peptide of RAS, angiotensin II, is produced by the action of angiotensin-converting enzyme (ACE) and acts through AT1 receptors [1]. The use of ACE inhibitors and AT1 receptor blockers is the universally recognized first-line strategy in the treatment of hypertension and cardiovascular disease [2].

Over the past 25 years, the classic understanding of RAS has undergone an extensive revision [3, 4]. The activities of different angiotensin-converting peptidases have been shown to produce several angiotensins that counterbalance the action of Ang II [1]. Further, several organs, tissues, and cells express all the components of RAS [5]. These local and intracellular RAS can regulate long-term biologic processes such as cell growth, proliferation, and tissue repair [1, 4, 5].

The newly expanded view of RAS describes endocrine, paracrine, autocrine, and intracrine functions and demonstrates that this peptidergic system regulates more diverse physiological phenomena than previously described [1, 4]. Moreover, the imbalance of RAS components has been associated with several chronic pathologies that go beyond cardiovascular and renal diseases [6], including proliferative (such as cancer) [7, 8] and degenerative disorders of the central nervous system (such as Alzheimer’s disease) [9, 10]. These diseases are primarily responsible for morbidity and mortality of adults in Western countries [11–13]. For this reason, there is great utility for RAS-modulating drugs and application of RAS components as diagnostic/prognostic biomarkers of chronic diseases [4, 7, 9, 14, 15].

Experimental evidence suggests an important influence of gonadal hormones on RAS, which could explain, in part, sex differences in arterial pressure and in the onset of cardiovascular diseases [3, 16]. Studies in animal models have shown that the expression of angiotensin-converting enzymes is regulated by estrogens and testosterone in several tissues [16]. These peptidases can be secreted to the extracellular space from several different tissues [17], and gender differences are reflected in body fluids [10]. Studies performed in children and young adults have described basal activity of ACE, as well as the maximum ACE inhibition after enalapril treatment, and found that they are significantly lower in women than in men, suggesting that estrogens and androgens could affect serum soluble ACE activity and lead to sexual differences in blood pressure [18]. Therefore, sex differences should be considered in the study of RAS components and the search for new serum biomarkers.

Here, we describe the activity of five crucial angiotensin-regulating peptidases (ACE, ACE2, aminopeptidase A (APA), aminopeptidase N (APN), and neutral endopeptidase (NEP) [1] in the serum of adult men and women. In our study, male and female individuals were described as good models of normal health and considered to be “control” subjects for chronic diseases. All participants were between 41 and 70 years old (typically, this is the age of onset of several cardiovascular and proliferative disorders and early stages of neurodegenerative diseases [11–13]), did not have any chronic pathology, and were not under any pharmacological treatment. Using this cohort, we sought to detect sex differences in RAS peptidase serum activity with aging.

## Methods

### Participants

Participants in this study comprised 118 healthy individuals with no history of any cardiovascular, proliferative, or central nervous system degenerative disease, ranging from 41–70 years old (60 males; age 56.58 ± 6.47 years; 58 females; age 54.47 ± 4.99 years). All participants underwent thorough medical examination, and we excluded all those who presented chronic pathologies involving drug treatment, such as estrogen replacement therapy, hormonal contraceptives or testosterone, or any significant neurologic, systemic, or psychiatric disorder that could cause cognitive impairment, as well as any who were pregnant or breastfeeding. All participants were informed of the extent of the study and signed an informed consent document approved by the Ethical and Scientific Committees (CEIC PGA-2).

### Sample preparation

Blood samples (10 mL) were collected by venipuncture into heparinized tubes after fasting overnight. Following centrifugation (1500 rpm, for 10 min), serum samples were separated and stored frozen at −80 °C until the analysis of renin–angiotensin system enzyme activity.

### Renin–angiotensin system-regulating enzyme activity

Enzyme activity in serum samples was measured by spectrofluorimetry in a microplate reader fluorometer FLUOstar OPTIMA (BMG Labtech). Angiotensin-converting enzyme (ACE/CD143: EC 3.4.11.1), angiotensin-converting enzyme 2 (ACE 2: EC 3.4.17.23), aminopeptidase A (APA/CD143: EC 3.4.11.7), aminopeptidase N (APN/CD13: EC 3.4.11.2), and neutral endopeptidase (NEP/CD10: EC 3.4.24.11) activity were measured. All samples were analyzed in triplicate and incubated at 37 ° C with their respective fluorogenic substrates in microplates for either 30 min (ACE, APA, APN, NEP/CD10) or 2 h (ACE2). Blanks were also included. The incubation times were based on preliminary assays to assess the linearity of the reaction over time as well as protein content. When introduced into the fluorimeter, specific emission and excitation filters for each substrate were selected. Each well was assayed with 10 flashes and the signals obtained were averaged. The value for the blank was discounted. The enzyme activity was expressed as units of enzyme activity per milliliter of serum (U/mL). One unit of activity is the amount of enzyme required to release 1 pmol of fluorescent product per minute. Specific inhibition assays for each substrate were performed to ensure appropriate measurement (86% or more inhibition accuracy was accepted).

#### ACE activity assay

Estimation of ACE activity was based on the fluorescence of the product generated upon hydrolysis by the enzyme of the substrate Abz-Gly-Phe(NO2) Pro (Bachem E2920). Fifty microliters of serum samples and 250 μL of substrate with 150 mM Tris-Hcl buffer, pH 8.3, plus 0.3 M NaCl were placed in a microplate reader and incubated as previously described. Excitation was at 355 nm and emission at 410 nm. The inhibition assay for ACE activity was performed with captopril (10 μM, Sigma).

#### ACE2 activity assay

The ACE2 activity assay was based on the fluorescence of the product generated upon hydrolysis by the enzyme of the substrate Abz-Ser-Pro-3-nitro-Tyr-OH (SPNPT; Bachem E2920). Reactions were initiated by adding 70 μL of the appropriate incubation mixture (substrate, 50 mM Tris-HCl buffer, pH 7.5, 2 μg BSA) to 30 μL of sample. After incubation, the fluorescence intensity was measured with an excitation filter at 355 nm and an emission filter at 410 nm. The inhibition assay for ACE2 activity was performed using DX600 (1 μM, Bachem).

#### APA and APN/CD13 activity assay

APA and APN/CD13 activity were measured using APA-β-naphthylamide as a substrate (Sigma N8381). Both were dissolved in 50 mM phosphate buffer. In the case of APA activity, 10 μL of serum were incubated with 190 μL of the mixture and 2 μg BSA, DTT, and CaCl2 were added to ensure that the reaction occurred. For APN activity, not only 2 μg of BSA but also the specific AlaAP inhibitor puromycin (40 μM) was added to discriminate between the AlaAP and APN/CD13 forms of total alanine aminopeptidase activity. Excitation was at 340 nm and emission at 410 nm. APA inhibition assay was performed using 1,10-phenanthroline (1,2 mM, Sigma) and APN/CD13 using leuhistin (0,05 mM, Sigma).

#### NEP/CD10 activity assay

The NEP/CD10 assay was performed by incubating 30-μL samples with 70 μL of a saturating concentration of N-dansyl-D-Ala-Gly-pNO2-Phe-Gly (DAGNPG, Sigma E39220). Captopril was added to discriminate between NEP activity and ACE forms. Excitation was at 340 nm and emission at 562 nm. NEP inhibition was performed using DL-thiorphan (Sigma).

### Statistical analyses

To compare sexes, the female and male groups were each subdivided into two groups, separated by the median value of the sample. All reported values represent the median of the individual determination ± interquartile range (IQR). Statistical analysis was performed using SPSS® Statistics version 21. The Kolmogorov–Smirnov test was used to test the normality distribution of the data. A Mann–Whitney *U* test was performed to detect differences between the sexes, as well as between younger (<55 years) and older (≥55 years) participants when variables showed a non-normal distribution.

Linear regression models using systolic and diastolic blood pressures as dependent variables and enzyme activities as independent variables, as well as sex and age as independent variables of influence, were applied. Additionally, a linear regression analysis between age and renin–angiotensin system-regulating enzyme activities in women and men was performed.

## Results

Descriptive parameters of 128 participants (all of them meeting inclusion criteria) are provided in Table 1. Men were found to have higher body mass indexes and higher blood pressure than women, as well as a lower body fat percentage. These differences were maintained among the specific age groups when a breakpoint of 55 years was selected to compare those less than or greater than 55 years of age (Table 2). A lack of changes in these parameters was observed in men when comparing the under 55 group to those over 55 years and in women over 55, presented a tendency toward higher systolic blood pressure (*P* = 0.078) compared to women under 55 years (Table 3).

**Table 1:** Descriptive statistics of the study population

	WOMEN			MEN			Mann–Whitney *U*	
	Median	IQR	N	Median	IQR	N	*Z*	*P* value
Age (years)	54.97	6.34	58	55.76	10.18	60	−1.55	0.121
BMI (kg/m^2^)	23.45	4.15	58	25.65	3.83	60	−3.51	<0.001
Body fat (%)	30.70	11.95	58	20.61	10.53	60	−5.61	<0.001
SBP (mmHg)	120.5	21.5	58	130.1	21.5	60	−3.34	0.001
DBP (mmHg)	80	5.25	58	80	3.75	60	−2.57	0.010

*Note*: *BMI* body mass index, *SBP* systolic blood pressure, *DBP* diastolic blood pressure

**Table 2:** Sex differences in BMI, body fat percentage, SBP, and DBP in younger (<55 years) and older (≥55 years) participants

		WOMEN			MEN			Mann–Whitney *U*	
		Median	IQR	N	Median	IQR	N	*Z*	*P* value
< 55 years	BMI (kg/m^2^)	23.5	4.4	28	25.4	4.5	31	−2.336	0.020
	Body fat (%)	30.1	14.9	28	20.2	10	31	−4.246	<0.001
	SBP (mmHg)	120	13	28	130	19	31	−2.783	0.005
	DBP (mmHg)	78.5	7	28	80	8	31	−0.005	0.047
> 55 years	BMI (kg/m^2^)	23.35	3.8	30	26.2	3.9	29	−2.664	0.008
	Body fat (%)	31.15	9.6	30	20.7	10.8	29	−3.735	0.000
	SBP (mmHg)	127	21	30	135	23	29	−1.803	0.071
	DBP (mmHg)	80	5	30	80	0	29	−1.683	0.092

*Note*: *BMI* body mass index, *SBP* systolic blood pressure, *DBP* diastolic blood pressure

**Table 3:** Effects of age (breakpoint >55 years) on BMI, body fat percentage, SBP, and DBP in all participants

		< 55 years			> 55 years			U Mann–Whitney	
		Median	IQR	N	Median	IQR	N	*Z*	*P* value
WOMEN	BMI (kg/m^2^)	23.5	4.4	28	23.35	3.8	30	−0.537	0.591
	Body fat (%)	30.1	14.9	28	31.15	9.6	30	−0.265	0.791
	SBP (mmHg)	120	13	28	127	21	30	−1765	0.078
	DBP (mmHg)	78.5	7	28	80	5	30	−0.459	0.646
MEN	BMI (kg/m^2^)	25.4	4.5	31	26.2	3.9	29	−0.059	0.953
	Body fat (%)	20.2	10	31	20.7	10.8	29	−0.792	0.429
	SBP (mmHg)	130	19	31	135	23	29	−1297	0.195
	DBP (mmHg)	80	8	31	80	0	29	−0.182	0.856

*Note*: *BMI* Body mass index, *SBP* Systolic blood pressure, *DBP* diastolic blood pressure

ACE, ACE2, NEP, APN, and APA serum enzymatic activities were measured in men and women (Table 4). Significantly lower serum APA activity levels were observed in men with respect to women; no changes were observed for ACE, ACE2, NEP, or APN.

**Table 4:** Renin–angiotensin system-regulating enzyme activities and sex differences

	TOTAL			WOMEN			MEN			Mann–Whitney *U*	
	Median	IQR	N	Median	IQR	N	Median	IQR	*N*	*Z*	*P* value
ACE	783.3	253.31	118	820.3	253.32	58	771.3	254.27	60	−1.281	0.2
ACE2	64.33	21.59	118	64.66	18.49	58	63.12	27.14	60	−0.350	0.726
NEP	15702	1724.59	118	15594	1272.12	58	15846	1974.44	60	−1.066	0.286
APN	10260	5417.84	118	10238	599.2	58	10436	4937.59	60	−0.711	0.477
APA	4143	1296.75	118	4345	1225.79	58	3946	991.35	60	−2.153	0.031

*Note*: *ACE* angiotensin-converting enzyme, *ACE2* angiotensin-converting enzyme-2, *NEP* neutral endopeptidase, *APN* aminopeptidase N, *APA* aminopeptidase A

Values are medians of peptidase activity recorded as pmol of units of peptidase (UP) per milliliter of serum

When we compared serum enzymatic activities separately in younger and older participants (breakpoint 55 years) (Table 5), significantly lower ACE and APA serum activity levels were observed in men with respect to women, but without changes in ACE2, NEP, or AP. However, in the case of younger participants, significantly higher NEP serum enzymatic activity was observed in men than in women, but without changes in ACE, ACE2, APN, or APA. In contrast, in the case of older participants, significantly lower ACE and APA serum activity was observed in men than in women, but without changes in ACE2, NEP, or APN.

**Table 5:** Sex differences in ACE, ACE2, NEP, APN, and APA serum activities in younger (<55 years) and older (≥ 55 years) participants

		WOMEN			MEN			Mann–Whitney *U*	
		Median	IQR	N	Median	IQR	N	*Z*	*P* value
< 55 years	ACE	820.3	249.77	28	841.4	281.02	31	−0.728	0.467
	ACE2	61.71	16.69	28	59.75	23.57	31	−0.032	0.975
	NEP	15474	1187.55	28	16203	2030.32	31	−2.017	0.044
	APN	9931	5046.74	28	11517	6916.75	31	−1.653	0.098
	APA	4304	1225.79	28	4281	1633.4	31	−0.121	0.903
> 55 years	ACE	815.9	272.13	30	744.4	201.43	29	−2.292	0.022
	ACE2	71.11	16.22	30	70,05	30.92	29	−0.378	0.706
	NEP	15703	1812.15	30	15344	1668.93	29	−0.562	0.574
	APN	10238	6688.67	30	9973	4001.23	29	−0.577	0.564
	APA	4361	1240.15	30	3820	623.97	29	−2.763	0.006

*Note*: *ACE* angiotensin-converting enzyme, *ACE2* angiotensin-converting enzyme-2, *NEP* neutral endopeptidase, *APN* aminopeptidase N, *APA* aminopeptidase A

The effects of age, with a breakpoint of 55 years, on ACE, ACE2, NEP, APN, and APA serum activity was measured in all the participants, as well as separately in men and women (Table 6). Significantly lower ACE and a trend toward lower APA (*P* = 0.063) serum enzymatic activity was detected in older men, but without changes in ACE2, NEP, or APN. In women, significantly higher ACE2 serum activity was observed in older women, but with no changes in ACE, NEP, APN, or APA. To evaluate the statistical association between age and serum aminopeptidase activities, linear regression models were applied separately in women and men (Table 7). These analyses indicated a positive association between ACE2 serum activity and age in women. Accordingly, a linear regression model using systolic and diastolic blood pressure as dependent variables and enzyme activities as independent variables, as well as sex and age as independent variables of influence, showed that age, sex, and ACE2 influence systolic blood pressure, while only sex influences diastolic blood pressure, across the continuum of age (Table 8).

**Table 6:** Effects of age (breakpoint >55 years) on ACE, ACE2, NEP, APN, and APA serum activities in women and men

		<55 years			≥ 55 years			Mann–Whitney *U*	
		Median	IQR	N	Median	IQR	N	*Z*	*P* value
WOMEN	ACE	820.3	249.77	28	815.9	272.13	30	−0.731	0.465
	ACE2	61.71	16.69	28	71.11	16.22	30	−2.169	0.030
	NEP	15474	1187.55	28	15703	1812.15	30	−0.965	0.335
	APN	9931	5046.74	28	10238	6688.67	30	−0.747	0.455
	APA	4304	1225.79	28	4361	1240.15	30	−0.327	0.744
MEN	ACE	841.4	281.02	31	744.4	201.43	29	−1.960	0.049
	ACE2	59.75	23.57	31	70.05	30.92	29	−1.007	0.313
	NEP	16203	2030.32	31	15344	1668.93	29	−1.013	0.311
	APN	11517	6916.75	31	9973	4001.23	29	−1.620	0.105
	APA	4281	1633.4	31	3820	623.97	29	−1.856	0.063

*Note*: *ACE* angiotensin-converting enzyme, *ACE2* angiotensin-converting enzyme-2, *NEP* neutral endopeptidase, *APN* aminopeptidase N, *APA* aminopeptidase A

**Table 7:** Linear regression anaysis between age and renin–angiotensin system-regulating enzyme activities in women and men

		*B* (95% CI)	β	*R* ^2^	*P* value
ACE	Women	5.979 (−5.408, 17.365)	0.139	0.019	0.297
	Men	−6.139 (−14.206, 1.927)	−0.196	0.038	0.133
ACE2	Women	0.959 (0.323, 1.595)	0.38	0.145	0.004
	Men	0.664 (−0.193, 1.521)	0.205	0.042	0.126
NEP	Women	14.87 (−48.08, 77.82)	0.063	0.004	0.638
	Men	−52.80 (−105.2, −0.402)	−0.256	0.066	0.048
APN	Women	162.9 (−68.22, 394.1)	0.185	0.034	0.163
	Men	−116.4 (−266.7, 33.78)	−0.2	0.040	0.126
APA	Women	51.75 (−16.21, 119.7)	0.2	0.040	0.133
	Men	−29.23 (−72.52, 14.06)	−0.175	0.031	0.182

*Note*: ACE angiotensin-converting enzyme, *ACE2* angiotensin-converting enzyme-2, *NEP* neutral endopeptidase, *APN* aminopeptidase N, *APA* aminopeptidase A

**Table 8:** Linear regression models including systolic blood pressure and diastolic blood pressure as dependent variables and enzyme activities as independent variables, as well as sex and age as independent variables of influence

Dependent variables	Predictors	B (95% CI)	β	R^2^	Adjusted *R* ^2^	SEE	*P* value
SBP	Model			0.478	0.228	15.263	<0.001
	Age (years)	110.54 (47.06, 174.03)	0.304				0.001
	Sex	−7.22 (−13.02,−1.43)	−0.221				0.015
	ACE2	27.99 (3.19, 82.78)	0.194				0.027
	Constant	−101.9 (−212.9, 9.22)					0.071
DBP	Model			0.225	0.051	6.685	<0.001
	Sex	−3.06 (−5.55,−0.57)	−0.221				0.015
	Constant	84.71 (80.78, 88.64)					<0.001

*Note*: *SBP* systolic blood pressure, *DBP* diastolic blood pressure, *ACE2* angiotensin-converting enzyme-2

## Discussion

Our results revealed sex differences in RAS serum enzymatic activities. Sex differences in soluble aminopeptidases, such as APA, have been described by other authors [19]. Moreover, it has been suggested that sex differences in blood pressure could be due to the effects of sex hormones on some of these peptidases, which are critical for the regulation of RAS [20]. In the present work, a critical range of age was selected for the inclusion of participants, all of whom were healthy middle-aged individuals, thus leading to a methodological improvement with respect to previous studies on this topic.

It has been proposed that sex differences in the regulation of arterial pressure by RAS could be due in part to a decrease in ACE serum activity in males, contrasting with a predominant ACE2 activity in females that could be lost in the post-menopausal period [16]. Sex differences in the incidence and evolution of cardiovascular disease and hypertension are also suggested to be due to the differential effects of sex steroids on critical components of RAS, which could possibly explain sex differences in blood pressure levels [21].

To detect specific physiological sex differences in the serum activities of RAS peptidases, we selected participants ranging from 41 to 70 years old, representing a period of time in which the onset of several cardiovascular and proliferative disorders and early stages of neurodegenerative diseases is most prevalent [11–13], who did not have any chronic pathology, and were not under any pharmacological treatment. Consequently, this sample was intended to constitute a specific ideal model, to avoid bias caused by concomitant pathologies and/or medications.

Estrogen decrease, whereas testosterone increases, ACE activity, and consequently Ang II levels [20]. Accordingly, in our study, men aged ≥55 years showed a significantly lower ACE activity with respect to women. Further, among men, a significant reduction in ACE activity was observed in older participants (>55 years), with respect to younger ones (<55 years). These results suggest a greater influence on ACE activity by testosterone, compared to estrogens.

Post-menopausal changes in estrogen levels are well known [22], as a consequence of the physiological decline in ovarian follicle numbers [23], leading to sleep and body temperature disturbances in older women [24]. Although andropausia has been described as a controversial concept [25], it has been suggested that androgen deficiency in older men has been overlooked in clinical settings [26]. In 2006, the Massachusetts Male Aging Study (MMAS) described a reduction in testosterone levels in 21% of men aged 55–59 years, 26% of those aged 60–69 years, and 31% of those aged 70–86 years [27]. In fact, late-onset hypogonadism (age-related testosterone deficiency) has been recently recognized as a clinical syndrome associated with low testosterone levels and alteration of health status [28, 29], and the possibility that subclinical hypogonadism in andropausal men could be a physiologic response of the hypothalamus has been proposed [30]. The breakpoint of 55 years selected for comparisons between groups of age in our study is relevant to these findings.

Pre-menopausal women have lower blood pressure with respect to post-menopausal women [31] and pre-andropausal men [32], indicating that a difference in cardiovascular risk could be mediated by RAS. In fact, androgens can stimulate RAS [33], and the sexually dimorphic pattern of hypertension in the spontaneously hypertensive rat is androgen-dependent, rather than estrogen-dependent [34]. Accordingly, serum peptidases, such as ACE, ACE2, NEP, APN, and APA, which are important elements of the RAS, were studied here because dysregulation of these enzymes has been associated with hypertension and cardiovascular risk [35]. Our results revealed sexual differences in these activities, with a different pattern in pre- and post-andropausal/post-menopausal subjects.

Sex differences in APA activity were observed in the selected subjects. More precisely, a significantly lower serum activity of this enzyme was found in men than in women. These results suggest lower production of angiotensin III in men, which exerts an important physiological regulatory action on cerebral circulation [36]. However, a different pattern of sexual differences was observed in participants ≥55 years of age. In this group, significantly lower APA and ACE serum activities were observed in men compared to women. Considering that angiotensin III promotes increased blood pressure when injected intracerebroventricularly [37], and taking into account that it has been reported that inhibitors of APA, the enzyme that produces angiotensin III, can act as antihypertensive drugs [38, 39]; these results could help explain the smaller changes in blood pressure usually observed in post-andropausal men, with respect to that observed in post-menopausal women.

The reduction in APA activity may be linked to a decrease in the production of angiotensin III, leading to a reduction in cerebral vasoconstriction. This could prevent the elevation of cerebral blood pressure, thus contributing to the decrease in cerebrovascular risk found in post-andropausal men compared to post-menopausal women. However, in participants <55 years old, although significantly higher NEP and APN serum activities were observed, a lack of changes in ACE, ACE2, and APA serum activities was observed in men, with respect to women. Notably, reduced APA could be linked to higher levels of angiotensin II, thus tending to increase blood pressure. Further, elevated NEP in younger men could increase angiotensin 1-7, reducing this parameter.

With respect to aging, these serum enzymatic activities presented sex differences in the activity patterns when it was observed between younger (<55 years) and older (≥55 years) participants. Thus, higher ACE2 activity was found in older women, whereas lower ACE and APA activities were observed in older men. Due to the fact that ACE2 activity can increase production of angiotensin 1-7, which leads to vasodilatatory, antiproliferative, and antifibrotic effects [40], and considering that antithrombotic effects have been recently proposed for the ACE2-angiotensin (1-7)-Mas receptor axis [41], the increase in ACE2 activity in the post-menopausal period in women could lead to a differential cardiovascular risk in men and women [42].

Although post-menopausal women are known to have an increased cardiovascular risk, this fact does not necessary contradict the beneficial cardiovascular effects of the increase in ACE2 activity in older women. Thus, it cannot be discarded that the ACE2-ang1-7-Mas axis could counterbalance negative cardiovascular effects of other age-related mechanisms. Further studies are needed to detect angiotensin peptide changes induced by aging in both sexes.

A limitation of the study could be that we lack data regarding alcohol consumption, cigarette smoking status, or physical activity habits, additional information that could influence RAS activity. However, we must emphasize that in meeting our inclusion criteria, the participants did not have any chronic pathology and were not under any pharmacological treatment, making this a reasonably good sample for this study.

## Conclusions

These results highlight sex differences in the aging pattern of renin–angiotensin system serum peptidases, suggesting a differential effect of aging in men, especially with respect to the breakpoint of andropausia, on the critical serum enzymatic activities of the RAS, which could correlate with sexual differences in cardiovascular risk.

## Abbreviations

ACE: Angiotensin-converting enzyme
ACE2: Angiotensin-converting enzyme-2
APA: Aminopeptidase A
APN: Aminopeptidase N
BMI: Body mass index
BSA: Bovine serum albumin
CEIC: Comité ético de investigación clínica de Euskadi
DBP: Diastolic blood pressure
DTT: Dithiothreitol
IQR: Interquartile range
MMAS: Massachusetts Male Ageing Study
NEP: Neutral endopeptidase
RAS: Renin–angiotensin system
SBP: Systolic blood pressure

## References

[1] Fyhrquist F, Saijonmaa O (2008). Renin-angiotensin system revisited. J Intern Med.

[2] Hamet P (2005). The renin-angiotensin system: where do we stand, and what is the future?. Am J Hypertens.

[3] Chappell MC, Marshall AC, Alzayadneh EM, Shaltout HA, Diz DI (2014). Update on the angiotensin converting enzyme 2-angiotensin (1-7)-MAS receptor axis: fetal programing, sex differences, and intracellular pathways. Front Endocrinol (Lausanne).

[4] Romero CA, Orias M, Weir MR (2015). Novel RAAS agonists and antagonists: clinical applications and controversies. Nat Rev Endocrinol.

[5] Paul M, Mehr AP, Kreutz R (2006). Physiology of local renin-angiotensin systems. Physiol Rev.

[6] Schmieder RE, Hilgers KF, Schlaich MP, Schmidt BM (2007). Renin-angiotensin system and cardiovascular risk. Lancet.

[7] George AJ, Thomas WG, Hannan RD (2010). The renin-angiotensin system and cancer: old dog, new tricks. Nature Rev Cancer.

[8] Wegman-Ostrosky T, Soto-Reyes E, Vidal-Millán S, Sánchez-Corona J (2015). The renin-angiotensin system meets the hallmarks of cancer. J Renin Angiotensin Aldosterone Syst.

[9] Wright JW, Kawas LH, Harding JW (2013). A role for the brain RAS in Alzheimer’s and Parkinson’s Diseases. Front Endocrinol (Lausanne).

[10] Puertas MC, Martínez-Martos JM, Cobo M, Lorite P, Sandalio RM, Palomeque T (2013). Plasma renin-angiotensin system-regulating aminopeptidase activities are modified in early stage Alzheimer’s disease and show gender differences but are not related to apolipoprotein E genotype. Exp Gerontol.

[11] Nichols M, Townsend N, Scarborough P, Rayner M (2013). Cardiovascular disease in Europe: epidemiological update. Eur Heart J.

[12] Siegel RL, Miller KD, Jemal A (2016). Cancer statistics, 2016. CA Cancer J Clin.

[13] Winblad B, Amouyel P, Andrieu S, Ballard C, Brayne C, Brodaty H (2016). Defeating Alzheimer’s disease and other dementias: a priority for European science and society. Lancet Neurol.

[14] Larrinaga G, Blanco L, Sanz B, Perez I, Gil J, Unda M (2012). The impact of peptidase activity on clear cell renal cell carcinoma survival. Am J Physiol Renal Physiol.

[15] Sanz B, Perez I, Beitia M, Errarte P, Fernández A, Blanco L (2015). Aminopeptidase N activity predicts 5-year survival in colorectal cancer patients. J Investig Med.

[16] Hilliard LM, Sampson AK, Brown RD, Denton KM (2013). The “His and Hers” of the renin-angiotensin system. Curr Hypertens.

[17] Rice GI, Jones AL, Grant PJ, Carter AM, Turner AJ, Hooper NM (2006). Circulating activities of angiotensin-converting enzyme, its homolog, angiotensin-converting enzyme 2, and neprilysin in a family study. Hypertension.

[18] Zapater P, Novalbos J, Gallego-Sandín S, Hernández FT, Abad-Santos F (2004). Gender differences in angiotensin-converting enzyme (ACE) activity and inhibition by enalaprilat in healthy volunteers. J Cardiovasc Pharmacol.

[19] Martínez JM, Ramírez MJ, Prieto I, Alba F, Ramírez M (1998). Sex differences and in vitro effects of steroids on serum aminopeptidase activities. Peptides.

[20] Ramírez-Expósito MJ, Martínez-Martos JM (2008). Hypertension, RAS and gender: what is the role of aminopeptidases?. Heart Fail Rev.

[21] Maric-Bilkan C, Manigrasso MB (2012). Sex differences in hypertension: contribution of the renin-angiotensin system. Gend Med.

[22] Burger HG (1996). The endocrinology of the menopause. Maturitas.

[23] Burger HG, Hale GE, Robertson DM, Dennerstein L (2007). A review of hormonal changes during the menopausal transition: focus on findings from the Melbourne Women’s Midlife Health Project. Hum Reprod Update.

[24] Murphy PJ, Campbell SS (2007). Sex hormones, sleep, and core body temperature in older postmenopausal women. Sleep.

[25] Pines A (2011). Male menopause: is it a real clinical syndrome?. Climacteric.

[26] Lee Y (2014). Androgen deficiency syndrome in older people. J American Association Nurse Practitioners.

[27] O’Donnell AB, Travison TG, Harris SS, Tenover JL, McKinlay JB (2006). Testosterone, dehydroepiandrosterone, and physical performance in older men: results from the Massachusetts Male Aging Study. J Clin Endocrinol Metab.

[28] Lunenfeld B, Mskhalaya G, Kalinchenko S, Tishova Y (2013). Recommendations on the diagnosis, treatment and monitoring of late-onset hypogonadism in men: a suggested update. Aging Male.

[29] Lunenfeld B, Mskhalaya G, Zitzmann M, Arver S, Kalinchenko S, Tishova Y, Morgentaler A (2015). Recommendations on the diagnosis, treatment and monitoring of hypogonadism in men. Aging Male.

[30] Corona G, Maseroli E, Rastrelli G, Sforza A, Forti G, Mannucci E, Maggi M (2014). Characteristics of compensated hypogonadism in patients with sexual dysfunction. J Sex Med.

[31] Dubey RK, Oparil S, Imthurn B, Jackson EK (2002). Sex hormones and hypertension. Cardiovasc Res.

[32] Reckelhoff JF (2001). Gender differences in the regulation of blood pressure. Hypertension.

[33] Kienitz T, Quinkler M (2008). Testosterone and blood pressure regulation. Kidney Blood Press Res.

[34] Chen YF, Meng QC (1991). Sexual dimorphism of blood pressure in spontaneously hypertensive rats is androgen dependent. Life Sci.

[35] Sánchez-Agesta Ortega R, de Saavedra-Alías JM A, Liébana-Cañada A, Sánchez-Muñoz B, Martínez-Martos JM, Ramírez-Expósito MJ (2013). Circulating aminopeptidase activities in men and women with essential hypertension. Curr Med Chem.

[36] Bausback HH, Churchill L, Ward PE (1988). Angiotensin metabolism by cerebral microvascular aminopeptidase A. Biochem Pharmacol.

[37] Reaux-Le Goazigo A, Iturrioz X, Fassot C, Claperon C, Roques BP, Llorens-Cortes C (2005). Role of angiotensin III in hypertension. Curr Hypertens Rep.

[38] Bodineau L, Frugière A, Marc Y, Claperon C, Llorens-Cortes C (2008). Aminopeptidase A inhibitors as centrally acting antihypertensive agents. Heart Fail Rev.

[39] Marc Y, Llorens-Cortes C (2011). The role of the brain renin-angiotensin system in hypertension: implications for new treatment. Prog Neurobiol.

[40] Santos RA, Ferreira AJ, Simões e Silva AC (2008). Recent advances in the angiotensin-converting enzyme 2-angiotensin (1-7)-Mas axis. Exp Physiol.

[41] Fraga-Silva RA, Da Silva DG, Montecucco F, Mach F, Stergiopulos N, da Silva RF, Santos RA (2012). The angiotensin-converting enzyme 2/angiotensin-(1-7)/Mas receptor axis: a potential target for treating thrombotic diseases. Thromb Haemost.

[42] Campesi I, Occhioni S, Tonolo G, Cherchi S, Basili S, Carru C, Zinellu A, Franconi F (2016). Ageing/menopausal status in healthy women and ageing in healthy men differently affect cardiometabolic parameters. Int J Med Sci.

